# Knowledge, Attitudes, Beliefs and Behaviors Regarding Fruits and Vegetables among Cost-Offset Community-Supported Agriculture (CSA) Applicants, Purchasers, and a Comparison Sample

**DOI:** 10.3390/nu11061320

**Published:** 2019-06-12

**Authors:** Karla L. Hanson, Leah C. Volpe, Jane Kolodinsky, Grace Hwang, Weiwei Wang, Stephanie B. Jilcott Pitts, Marilyn Sitaker, Alice S. Ammerman, Rebecca A. Seguin

**Affiliations:** 1 Division of Nutritional Sciences, Cornell University, Ithaca, NY 14853, USA; lmc267@cornell.edu (L.C.V.); gwh77@cornell.edu (G.H.); rs946@cornell.edu (R.A.S.); 2 Department of Community Development and Applied Economics, University of Vermont, Burlington, VT 05405, USA; jane.kolodinsky@uvm.edu (J.K.); wwang@uvm.edu (W.W.); 3 Department of Public Health, Brody School of Medicine, East Carolina University, Greenville, NC 27834, USA; jilcotts@ecu.edu; 4 The Evergreen State College, Ecological Agriculture and Food System, Olympia, WA 98505, USA; msitaker@gmail.com; 5 Gillings School of Global Public Health and School of Medicine, University of North Carolina at Chapel Hill, Chapel Hill, NC 27599, USA; alice_ammerman@unc.edu

**Keywords:** fruits and vegetables, dietary quality, local food, community-supported agriculture (CSA), low-income

## Abstract

Community-supported agriculture (CSA) participation has been associated with high fruit and vegetable (FV) consumption, which may be due to better access to FV for CSA purchasers, or to positive knowledge, attitudes, and beliefs (KAB) regarding healthy eating among CSA applicants. The objective of this study was to examine KAB and consumption, in association with application to a cost-offset CSA (CO-CSA) program, and with CO-CSA purchase among applicants. We conducted a cross-sectional survey of CO-CSA applicants and a comparison sample in August 2017. All respondents were English-reading adults with a child 2–12 years old and household income of ≤185% of the federal poverty level. Among CO-CSA applicants, some were CO-CSA purchasers (*n* = 46) and some were not (*n* = 18). An online comparison sample met equivalent eligibility criteria, but had not participated in CSA for three years (*n* = 105). We compared CO-CSA applicants to the comparison sample, and compared purchasers and non-purchaser sub-groups, using Mann-Whitney *U* tests and chi-square analysis. CO-CSA applicants reported better knowledge, self-efficacy, home habits, and diet than the comparison sample. Among applicants, CO-CSA purchasers and non-purchasers had equivalent KAB, but children in purchaser households had higher FV consumption than in non-purchaser households (4.14 vs. 1.83 cups, *p* = 0.001). Future research should explore associations between CO-CSA participation and diet using experimental methods.

## 1. Introduction

Fruits and vegetables (FV) provide high concentrations of dietary fiber, vitamins, minerals, and antioxidants, have low energy density [1], and are recommended as an important component of a healthy diet [2]. FV consumption is associated with reduced risk of cardiovascular disease, cancer, and mortality among adults [3,4,5,6]. Although the link between FV intake and health outcomes during childhood is less clear [7,8], childhood eating habits persist into adulthood, and life-long healthy eating can prevent some adult chronic disease [9]. FV consumption in the US is less than half the recommended amount [10,11], and even lower among adults and children in households with low income [12,13] and food insecurity [14]. Thus, strategies to increase FV consumption among at-risk adults and children are important to improve health.

Personal and economic factors both influence FV consumption. A systematic review shows knowledge about FV, attitudes toward the taste of FV, and self-efficacy are consistently associated with FV consumption for adults [15]. In low-income communities, however, intake of FV is mediated by poorer access to quality FV at affordable prices [16,17,18,19]. Improved accessibility and affordability of FV has the potential to enhance dietary quality for these at-risk individuals. A systematic review of economic data suggests that even a 10% reduction in the price of FV could stimulate a 4–10% increase in produce purchases [20], and most experimental research shows that incentives for FV purchase increase their consumption [21,22,23,24].

Community-supported agriculture (CSA) is a partnership between a local farm and customer in which the customer pays the farm a set price before the growing season for a ‘share’ of the farm’s harvest. As such, it is a direct-to-consumer distribution model [25,26]. Consumers generally pick up their shares at a set location, day, and time each week. Some farms deliver a box of seasonal items, and other farms allow customers to select among seasonal items. CSA farms have the potential to improve access to FV, because they can provide higher quality produce at lower prices than grocery stores [27,28]. However, concerns about affordability hinder CSA participation and retention [29,30,31,32,33,34]. Some programs offer subsidized or cost-offset CSA (CO-CSA) shares at reduced prices to low-income households in order to facilitate participation [35], but few studies have explored the experience of low-income families within CO-CSA [34,36,37,38,39,40,41]. One observational study reported that adults and children in low-income households that participated in CO-CSA had FV intake significantly higher than the US median, and more than 80% met recommendations for vegetable intake, compared to less than 10% in the general population [42]. One interpretation of those results suggests a hypothesis that CO-CSA purchases enhance FV access, which facilitates higher FV consumption. In other words, caregivers and children in CO-CSA households eat more FV because of the enhanced availability and affordability of FV due to CSA. An alternative interpretation suggests a hypothesis that CO-CSA attracts participants who already possess positive knowledge, attitudes, beliefs (KAB), and home habits with respect to healthy eating. These positive KAB and habits motivate them to overcome barriers to FV access and maintain healthy eating habits, and attract them to CO-CSA.

This study had dual objectives. First, we explored associations between KAB and diet among CO-CSA applicants and non-applicants for evidence to support or refute the hypothesis of positive selection into CO-CSA. Due to limited funding for cost-offsets in the studied program, applicants were offered CO-CSA purchase on a first-come, first-served basis and not all applicants were offered a CO-CSA. Therefore, our second objective was to examine associations between KAB and diet among applicants who purchased a CO-CSA and those who did not for evidence to support or refute the hypothesis of CO-CSA expanding access to FV, which may result in the higher FV consumption previously observed. At the time of data collection, the purchaser sub-group received weekly assortments of fresh, whole fruits and vegetables through the CO-CSA, whereas, the non-purchaser group did not. We triangulated these results and discussed whether the hypothesis of FV accessibility or the hypothesis of positive selection better characterized the pattern of associations observed.

## 2. Materials and Methods

This paper reports on a cross-sectional on-line survey of adult applicants to a 50% CO-CSA program for low-income households, and a comparison sample that did not participate in any CSA. Institutional review boards at Cornell University (#1501005266) and the University of Vermont (#15640 & #16177) approved the study protocol, and consent was obtained electronically.

### 2.1. Participants/Setting

The CO-CSA applicant sample included English-reading adults, with household incomes of ≤185% of the US federal poverty level, who had applied to a CO-CSA program in the state of Vermont, and who reported at least one child 2–12 years of age in the household. Some of the CO-CSA applicant sample had been recruited in August 2015 or August 2016 as part of a longitudinal survey, the details of which are reported elsewhere [42]. In August 2017, all longitudinal survey respondents were invited to participate again (*n* = 80), and new applicants to the CO-CSA who met eligibility criteria were also invited to participate (*n* = 60). The CO-CSA applicant sample (*n* = 64) pooled 33 longitudinal survey respondents (41% response rate) and 31 new respondents (52% response rate). Respondents were compensated $25. Applicants were offered cost-offsets on a first-come, first-served basis until program funds were exhausted, resulting in two applicant sub-groups: CO-CSA purchasers, who received a cost-offset and were confirmed to have purchased a summer 2017 CSA share (*n* = 46) and non-purchasers, who were not offered a cost-offset and were confirmed to have not purchased any summer 2017 CSA shares (*n* = 18).

Also in August 2017, a comparison sample was recruited from the Qualtrics panel system (Qualtrics, Provo, UT), which consists of a large number of community-based individuals who provide online data for which they earn points toward compensation. Potential comparison sample participants received an email from Qualtrics inviting them to respond to a screening questionnaire in order to assess eligibility. The comparison sample was selected by screening participants for comparable attributes such as gender (female), household income (≤185% of the US federal poverty level), household composition (household that includes a child 2–12 years of age), and regional geography (New England region), and participants also could not have participated in any CSA in the past three years (*n* = 105). Given the small population of Vermont, comparison sample members were recruited from all states in New England—Connecticut (12.4%), Maine (18.1%), Massachusetts (34.3%), New Hampshire (19.0%), Rhode Island (9.5%) and Vermont (6.7%).

We had sufficient power to detect (*β* = 0.8, *α* = 0.05) a small difference in FV consumption (one-third cup) between CO-CSA applicant and comparison samples, and a large difference (1.8 cups) between CO-CSA applicant sub-groups of purchasers and non-purchasers.

### 2.2. Measures

Personal factors included KAB regarding nutrition and cooking. Caregiver’s knowledge of FV recommendations was assessed by two original questions that asked about knowledge of FV recommendations in cups and again based on plate coverage. Responses were dichotomized into ‘correct’ (5+ cups FV/day, or ≥½ dinner plate FV = 1) or not (0) [2].

Measures of caregiver attitudes and self-efficacy were developed by Condrasky and colleagues (2011) using factor analysis with a sample of 245 US parents and cooks, and all three scales reported high reliability [43]. The Negative Cooking Attitudes scale was the mean response to four negative statements about cooking (Cronbach’s *α* = 0.92 in the current study). Responses ranged from strongly disagree (1) to strongly agree (5). Higher scores indicated greater negativity toward cooking. Belief in one’s own capabilities was measured as two types of self-efficacy. The Cooking Techniques and Meal Preparation Self-Efficacy scale (Cronbach’s *α* = 0.92 in the current study) assessed confidence in 14 food preparation skills (e.g., knife skills, roasting). The Self-Efficacy for Eating/Cooking FV scale (Cronbach’s *α* = 0.83 in the current study) queried four items relating to fruits and vegetables (e.g., eating FV with every meal, as a snack) [43]. Both scales used the mean of all components, and ranged from ‘not at all confident’ (1) to ‘extremely confident’ (5).

General nutrition beliefs were assessed using the General Nutrition Knowledge Belief Score, which includes 11 healthy eating statements (e.g., eat plenty of FV, use sugar in moderation) selected by Beydoun and colleagues using principal components analysis in a representative US sample of 4,356 adults [44]. The scale was the mean of responses that ranged from ‘not at all important’ (1) to ‘very important’ (4) (Cronbach’s *α* = 0.75 in the current study). Home habits were reflected in availability and accessibility of FV in the home. FV availability was assessed by three subjective frequency items (e.g., FV available, vegetables served at meals, fruit served for dessert), and FV accessibility by four items (e.g., cut-up vegetables in the fridge, fresh fruit on the counter) [45]. Both scales use the mean of all components, and range from ‘hardly ever’ (1) to ‘often’ (4). Although used in prior studies, neither scale has been validated to date.

Dietary data were obtained for an adult and one randomly-selected child aged 2–12 years in each household. In households in which there was more than one child aged 2 to 12 years, one child was randomly selected by the computer for dietary reporting. Caregivers were instructed to report dietary intake for younger children, and to report with children seven and older in order to improve accuracy [46]. Daily FV consumption was assessed with the National Cancer Institute All-Day Fruit and Vegetable Screener (FVS). The FVS collected information on the frequency and usual portion size for nine fruit and vegetable components (100% juice, fruit, lettuce salad, fried potatoes, other white potatoes, cooked dried beans, other vegetables, tomato sauce, and vegetable soup) in the past month [47]. The FVS is considered a moderately reliable measurement tool for adults (test/re-test *r* = 0.65) [48], and is moderately correlated with FV consumption from 24-hour recalls with men (*r* = 0.66) and women (*r* = 0.51) [47]. However, some evidence suggests the FVS may overestimate FV consumption, particularly among women [47,49], and it has not yet been validated in children. We formatted online questions about usual portion size to include corresponding photographs in an effort to reduce reporting bias [50]. The product of frequency and portion was converted into daily 2005 MyPyramid cup equivalents for each FVS component, and all products were summed into total FV consumption. One auxiliary FV consumption variable excluded potatoes and dried beans to better align with World Health Organization FV recommendations, which do not include dried beans and starchy tubers such as potatoes [51], and one variable excluded fruit juice to better align with the government’s Dietary Guidelines for Americans, which emphasize whole fruits over fruit juice [2].

Consumption of sweets, salty snacks, and sugar-sweetened beverages (SSBs) were collected using 15 questions about consumption in the past week, which were themselves selected from the second version of the beverage and snack questionnaire (BSQ2) [52,53]. The BSQ2 provided seven ordinal response categories from ‘never or <once per week’ to ‘4+ times per day,’ all of which were subsequently converted into times per month. The BSQ2 asked about frequency of consumption ‘at school’ and ‘not at school’ for children, and did not qualify location for adults. Frequency of consumption of ‘total sweets’ summed responses for five types of sweets, ‘total salty snacks’ summed three salty items, and ‘SSBs’ summed seven beverages that contain added sugars. These BSQ2 measures were validated in a sample of 46 ethnically-diverse young adolescents (test/re-test *r* = 0.72 to 0.77; correlation with food diaries *r* = 0.63 to 0.71) [52,53].

Household characteristics included: (1) number of adults and number of children calculated from a household roster; (2) household food security classified by the six-item short form of the United States Department of Agriculture (USDA) Food Security Survey Module (FSSM), with a 30-day reference period [54]; and (3) participation in the past month in the Supplemental Nutrition Assistance Program (SNAP) and, separately, the Special Supplemental Nutrition Program for Women, Infants, and Children (WIC). Respondent and child age and sex were obtained from the household roster. Fifteen nominal choice options (including ‘other’) recorded respondent race, but only the percentages of white and Hispanic are reported, due to low response in other categories. Education is reported in the five ordinal categories.

### 2.3. Analysis

All measures were summarized and compared for the CO-CSA applicant and comparison samples, and subsequently for applicant subgroups of purchasers and non-purchasers. All analyses were performed with SPSS version 23 software (IBM Corp., Armonk, NY). Sample characteristics were summarized with percentages and means, and compared across samples using chi-square analyses and *t* tests. Measures of attitudes, self-efficacy, and diet were tested for normality, and the majority had skewness statistics > 1.0. Therefore, these variables were summarized with medians and compared across samples using Mann-Whitney *U* tests. Small amounts of missing data (<10%) are noted in footnotes to the tables. Results were considered statistically significant when *p* < 0.05.

## 3. Results

The CO-CSA applicant and comparison samples had some similarities: most had two children, households received SNAP and WIC benefits at similar rates, and almost all respondents were white (92%; Table 1). However, in contrast to the comparison sample, more CO-CSA applicant households were food secure (62.3 vs. 26.5% were food secure, *p* < 0.001). In addition, CO-CSA applicants were slightly less often female, were older, and had greater educational attainment than the comparison sample (67.2 vs. 22.9% had a college degree or more, *p* < 0.001). There were no significant differences in characteristics between purchaser and non-purchaser applicant sub-groups.

Relative to the comparison sample, CO-CSA applicants had greater knowledge of FV recommendations, higher self-efficacy for cooking and meal preparation, and healthier home habits (Table 2). In particular, applicants had high self-efficacy for cooking and meal preparation (median of 4.2 on a 5-point scale). However, the two samples had equivalent general nutrition beliefs and the CO-CSA applicants had greater negativity toward cooking relative to the comparison group.

Adult dietary quality among CO-CSA applicants was better than in the comparison group. Applicants ate more FV (4.1 vs. 2.0 cups/day, *p* < 0.001), a difference that was maintained without potatoes and dried beans, or juice. Applicants also had lower frequency of consumption of sweets (15.1 vs. 21.5 times/month, *p* = 0.001), salty snacks (8.6 vs. 15.1 times/month, *p* < 0.001), and SSBs (4.3 vs. 35.5 times/month, *p* < 0.001) than the comparison sample. Children of CO-CSA applicants also had better dietary quality relative to the comparison sample, as they ate more FV when the measure excluded potatoes and dried beans (3.59 vs. 1.97, *p* = 0.041) or juice (3.3 vs. 1.6 cups/day, *p* < 0.001). Children of applicants also less frequently consumed sweets (15.1 vs. 22.6 times/month, *p* = 0.002), salty snacks (12.9 vs. 21.5 times/month, *p* < 0.001), and SSBs (0.0 vs. 30.1 times/month, *p* < 0.001).

Fewer differences were observed between CO-CSA applicant sub-groups. Purchasers had higher self-efficacy for eating and cooking FV than did non-purchasers (3.8 vs. 2.5, *p* = 0.035), yet had equivalent dietary quality. For children, however, those who lived in a purchaser household had higher FV consumption than children living in non-purchaser households (4.1 vs. 1.8 cups/day, *p* = 0.001), a difference that was maintained without potatoes and dried beans, or juice.

## 4. Discussion

Prior research has noted higher FV consumption among CSA participants [25,31,34,42,55,56,57,58,59,60], including two that examined CO-CSA participants from low-income households [34,42] and one that studied their children [42]. We hypothesized that higher observed FV consumption could be due either to positive selection into CSA, or to a positive effect of the CSA on FV availability in the household and consumption by household members. We discuss our findings in relationship to each of these hypotheses below.

The hypothesis of positive selection into CSA among low-income households was supported by greater observed knowledge and self-efficacy related to healthy eating and food preparation among CO-CSA applicants than in the comparison sample. Self-efficacy for cooking and meal preparation was high (4.2 on a 5-point scale), and comparable to that observed in a sample of CSA participants at a federally qualified health center (4.5 on a 5-point scale) [61] and consistent with a pilot study in which most CO-CSA participants reported that they knew how to prepare the FV in their CSA share [37]. CO-CSA applicants, however, did not always hold more positive attitudes and beliefs than their comparison counterparts. In particular, CO-CSA applicants had poor cooking attitudes relative to the comparison adults. Since attitudes are developed partly through experiences [62], more experience preparing fresh, whole produce like that provided in CO-CSA shares may help to explain these more negative attitudes. These negative cooking attitudes, however, must be viewed alongside their greater self-efficacy for meal preparation, the development of which did not appear impeded by their attitudes.

Applicants to CO-CSA and their children also had better indicators of dietary quality—greater consumption of FV and less frequent consumption of processed snacks and SSBs—and more often lived in food-secure households than comparison households. However, CO-CSA applicants were also older and had more education than the comparison sample. Prior research has reported CSA participation is limited to primarily middle-income and upper-income households [25,29,56,58,63,64,65]. These observed differences in educational attainment between applicants and non-applicants, all of whom reside within low-income households, suggests financial barriers to CSA purchase may not be the only socio-economic differences between applicants and non-applicants. Furthermore, age and education are known to be associated positively with KAB that support healthy eating [66] and with dietary quality [67]. In these cross-sectional data from a relatively small sample, it is not possible to explore the complex inter-relationships between age, education, KAB, dietary quality, and food security, nor to determine the direction of any causality. Research is needed to explore the dynamic pathways among these constructs and their impact on diet-related disease.

The second hypothesis—that CSA participation has a positive effect on FV availability and consumption—was supported by greater self-efficacy for eating and cooking FV among CO-CSA applicant-purchasers than among applicant non-purchasers. Further, children in applicant-purchaser households had higher FV consumption relative to children in non-purchaser households. Within the applicant sample, purchasers and non-purchasers were otherwise similar in KAB and diet. To our knowledge, no prior research has examined CO-CSA applicant-purchasers relative to non-purchaser applicants. Data were collected in August, amid the summer CSA season, when shares include many of the summer FV preferred by low-income caregivers and their children [33], which may have contributed to the magnitude of the differences in children’s FV consumption that we observed. Because resources to support the CO-CSA were limited and offsets were awarded on a first-come first-served basis, and because almost all KABs were equivalent for purchaser and non-purchaser sub-groups, selection bias into the purchaser sub-group is an unlikely explanation for differences observed between CO-CSA purchasers and non-purchasers. However, all results in support of this hypothesis emerged from tests with low power.

These analyses have several limitations. First, these cross-sectional data do not allow rigorous examination of any causal link between CO-CSA participation and dietary quality. Second, the inclusion of some CO-CSA applicants who participated in a longitudinal study regarding CO-CSA may have biased upwards estimates of KAB and self-efficacy with respect to FV consumption in the applicant sample. Third, although selection of adults into the comparison group used similar eligibility criteria, they were not comparable to CO-CSA applicants who were older, more educated, and more often lived in food-secure households. Fourth, the reliability of the FVS as a measure of total FV consumption is only moderate, but this potential measurement error is not expected to differ for the applicant and comparison samples. Fifth, CO-CSA purchasers may have experienced more social desirability bias in reporting diet than the comparison sample and the non-purchaser sub-group, which may have exaggerated observed differences in dietary quality. Finally, sample sizes were too small to permit exploration of observed associations in a multivariate context that controlled for key sample differences such as age, educational attainment, or self-efficacy for eating and cooking FV.

## 5. Conclusions

Evidence from this study supports both the hypothesis of the positive selection into CO-CSA and the hypothesis of the positive effect of CO-CSA on FV consumption. CO-CSA applicants were older, more educated adults with more positive KAB and healthier diet habits for themselves and their children. This suggests that broader recruitment into CO-CSA programs may need to include targeted efforts to effectively engage with households that apply having less educational attainment, lower knowledge and self-efficacy, and fewer healthy home habits. Among these applicants, however, CO-CSA purchasers had higher self-efficacy, and their children had higher FV consumption relative to applicants who were not purchasers. It is not clear why CO-CSA purchase was associated with better FV consumption among children and not adults. Future research should explore associations between CO-CSA participation and FV consumption using an experimental design with longitudinal data, and a larger sample that will allow for multivariate statistical techniques to consider the mediating pathways between KAB, CO-CSA, FV access, and dietary quality. CO-CSA programs have the potential to enhance FV access and consumption among low-income families, although they currently attract more adults with enhanced KAB and self-efficacy with respect to healthy eating.

## Figures and Tables

**Table 1:** Characteristics of low-income applicants to a CO-CSA program (*n* = 64) and a comparison sample (*n* = 105).

CHARACTERISTICS	CO-CSA Applicants	Comparison Sample	*p*	Sub-Groups of CO-CSA Applicants		
	(*n* = 64) ^1^	(*n* = 105) ^2^		CO-CSA Purchasers (*n* = 46)	Non-Purchasers (*n* = 18)	*p*
**HOUSEHOLD**						
**Number of adults**, %						
1 adult	37.5	22.9	0.003	34.8	44.4	0.775 ^4^
2 adults	56.3	51.4		58.7	50	
3+ adults	6.3	25.7		6.5	5.6	
**Number of children**, %						
1 child	31.3	29.5	0.175	26.1	44.4	0.188 ^4^
2 children	50	38.1		56.5	33.3	
3 children	15.6	21.9		13	22.2	
4+ children	3.1	10.5		4.3	0	
**Food Security Status**, %						
Food Secure	62.3	26.5	<0.001	63.6	58.8	0.186 ^4^
Low Food Security	21.3	29.4		25	11.8	
Very Low Food Security	16.4	44.1		11.4	29.4	
**SNAP in past month**, %	48.4	53.8	0.496	55.6	29.4	0.066
**WIC in past month**, %	24.2	35.2	0.136	22.2	29.4	0.740 ^3^
**CAREGIVER**						
**Female**, %	90.5	100	0.002 ^3^	88.9	94.4	0.664 ^3^
**Age, mean** (SE)	39.0 (0.9)	32.3 (0.6)	<0.001	39.2 (1.1)	38.5 (1.6)	0.733
**White race**, %	91.7	92.4	1.000 ^4^	93	88.2	0.616 ^3^
**Hispanic/Latino**, %	3.3	9.5	0.214 ^3^	4.5	0	1.000 ^3^
**Education**, %						
High school or less	6.6	30.5	<0.001	6.8	5.9	0.978 ^4^
Technical/vocational	0	13.3		0	0	
Some college	26.2	33.3		25	29.4	
College graduate	32.8	21.9		34.1	29.4	
Graduate/professional	34.4	1		34.1	35.3	
**CHILD**						
**Female**, %	43.8	47.6	0.625	41.3	50	0.528
**Age, mean** (SE)	7.7 (0.4)	6.1 (0.3)	0.003	7.5 (0.5)	8.5 (0.9)	0.293

CO-CSA, cost-offset community supported agriculture; SNAP, the Supplemental Nutrition Assistance Program; WIC, the Special Supplemental Nutrition Program for Women, Infants, and Children. Differences between groups were tested using *t* tests and Pearson’s Chi-square analysis unless otherwise noted. ^1^ CO-CSA applicant sample size varied from 60–64 due to missing data. ^2^ Comparison sample size varied from 91–105 due to missing data. ^3^ Differences between groups were tested using Fishers’ exact tests due to small expected cell counts. ^4^ Difference between groups were tested using likelihood ratio tests due to small expected cell counts.

**Table 2:** Personal factors and dietary quality among low-income applicants to a CO-CSA program (*n* = 64) and a comparison sample (*n* = 105).

	CO-CSA Applicants (*n* = 64) ^1^	Comparison Sample (*n* = 105) ^2^	*p*	Sub-Groups of CO-CSA Applicants		
				CO-CSA Purchasers (*n* = 46)	Non-purchasers (*n* = 18)	*p*
**PERSONAL FACTORS**	**%**	**%**		**%**	**%**	
**Knowledge**						
5+ cups/day of FV recommended	51.7	24.8	0.001	53.5	46.7	0.649
½ of dinner plate should be FV	91.4	42.9	<0.001	90.7	93.3	1.000 ^3^
**Attitudes**	**median**	**median**		**median**	**median**	
***Negative*** cooking attitudes (higher numbers indicate poorer attitudes)	2.5	2.25	0.042	2.5	3.25	0.269
**Beliefs**						
General nutritional beliefs	3.09	3.18	0.494	3.09	3.09	0.682
Self-efficacy:						
for cooking and meal preparation	4.18	3.79	<0.001	4.14	4.21	0.929
for eating and cooking FV	3.5	3.5	0.33	3.75	2.5	0.035
**Home habits**						
Availability of FV in the home	3.33	3	<0.001	3.67	3.33	0.062
Accessibility of FV in the home	3.5	3	0.001	3.75	3.5	0.104
**CAREGIVER DIET**						
Total FV, cups/day	4.09	1.97	<0.001	4.09	4.11	0.485
Total FV (without potatoes & dried beans), cups/day	3.71	1.52	<0.001	3.71	3.72	0.523
Total FV (without juice), cups/day	3.97	1.61	<0.001	3.97	3.95	0.548
Ate sweets, times/month	15.05	21.5	0.001	17.2	10.75	0.136
Ate salty snacks, times/month	8.6	15.05	<0.001	8.6	8.6	0.649
Drank SSBs, times/month	4.3	35.48	<0.001	4.3	8.6	0.219
**CHILD DIET**						
Total FV, cups/day	3.6	2.22	0.093	4.14	1.83	0.001
Total FV (without potatoes & dried beans), cups/day	3.59	1.97	0.041	3.86	1.6	0.001
Total FV (without juice), cups/day	3.28	1.6	<0.001	3.74	1.4	0.002
Ate sweets not at school, times/month	15.05	22.58	0.002	17.2	15.05	0.621
Ate salty snacks not at school, times/month	12.9	21.5	<0.001	15.05	9.68	0.229
Drank SSBs not at school, times/month	0	30.1	<0.001	0	6.45	0.356

CO-CSA, cost-offset community supported agriculture; FV, fruits and vegetables; SSBs, sugar-sweetened beverages. Differences between groups were tested using Pearson’s Chi-square analysis for dichotomous measures of knowledge (unless otherwise noted), and with Mann-Whitney *U* tests for all other measures. ^1^ CO-CSA applicant sample size varied from 53–64 due to missing data. ^2^ Comparison sample size varied from 102–105 due to missing data. ^3^ Differences between groups were tested using Fishers’ exact tests due to small expected cell counts.

## References

[1] World Health Organization Diet, Nutrition, and the Prevention of Chronic Diseases: Report of a Joint WHO/FAO Expert Consultation.

[2] US Department of Health and Human Services and US Department of Agriculture (2015). 2015–2020 Dietary Guidelines for Americans.

[3] Ness A.R., Powles J.W. (1997). Fruit and vegetables, and cardiovascular disease: A review. Int. J. Epidemiol..

[4] Reddy K.S., Katan M.B. (2004). Diet, nutrition and the prevention of hypertension and cardiovascular diseases. Public Health Nutr..

[5] Steinmetz K.A., Potter J.D. (1996). Vegetables, fruit, and cancer prevention: A review. J. Am. Diet. Assoc..

[6] Aune D., Giovannucci E., Boffetta P., Fadnes L.T., Keum N., Norat T., Greenwood D.C., Riboli E., Vatten L.J., Tonstad S. (2017). Fruit and vegetable intake and the risk of cardiovascular disease, total cancer and all-cause mortality-a systematic review and dose-response meta-analysis of prospective studies. Int. J. Epidemiol..

[7] Ledoux T., Hingle M., Baranowski T. (2011). Relationship of fruit and vegetable intake with adiposity: A systematic review. Obes. Rev..

[8] Marshall S., Burrows T., Collins C. (2014). Systematic review of diet quality indices and their associations with health-related outcomes in children and adolescents. J. Hum. Nutr. Diet..

[9] Craigie A., Lake A., Kelly S., Adamson A., Matherse J. (2011). Tracking of obesity-related behaviours from childhood to adulthood: A systematic review. Maturitas.

[10] Kim S.A., Moore L.V., Galuska D., Wright A.P., Harris D., Grummer-Strawn L.M., Merlo C.L., Nihiser A.J., Rhodes D.G. (2014). Vital signs: Fruit and vegetable intake among children—United States, 2003–2010. Morb. Mortal. Wkly. Rep..

[11] Moore L.V., Dodd K.W., Thompson F.E., Grimm K.A., Kim S.A., Scanlon K.S. (2015). Using Behavioral Risk Factor Surveillance System data to estimate the percentage of the population meeting US Department of Agriculture food patterns fruit and vegetable intake recommendations. Am. J. Epidemiol..

[12] Bowman S. (2007). Low economic status is associated with suboptimal intakes of nutritious foods by adults in the National Health and Nutrition Examination Survey 1999–2002. Nutr. Res..

[13] Lallukka T., Pitkaniemi J., Rahkonen O., Roos E., Laaksonen M., Lahelma E. (2010). The association of income with fresh fruit and vegetable consumption at different levels of education. Eur. J. Clin. Nutr..

[14] Hanson K.L., Connor L.M. (2014). Food insecurity and dietary quality in US adults and children: A systematic review. Am. J. Clin. Nutr..

[15] Guillaumie L., Godin G., Vezina-Im L.A. (2010). Psychosocial determinants of fruit and vegetable intake in adult population: A systematic review. Int. J. Behav. Nutr. Phys..

[16] Franco M., Roux A.V.D., Glass T.A., Caballero B., Brancati F.L. (2008). Neighborhood characteristics and availability of healthy foods in Baltimore. Am. J. Prev. Med..

[17] Hendrickson D., Smith C., Eikenberry N. (2006). Fruit and vegetable access in four low-income food deserts communities in Minnesota. Agric. Hum. Values.

[18] Larson N.I., Story M.T., Nelson M.C. (2009). Neighborhood environments: Disparities in access to healthy foods in the US. Am. J. Prev. Med..

[19] Zenk S.N., Odoms-Young A.M., Dallas C., Hardy E., Watkins A., Hoskins-Wroten J., Holland L. (2011). You have to hunt for the fruits, the vegetables: Environmental barriers and adaptive strategies to acquire food in a low-income African American neighborhood. Health Educ. Behav..

[20] Andreyeva T., Long M.W., Brownell K.D. (2010). The impact of food prices on consumption: A systematic review of research on the price elasticity of demand for food. Am. J. Public Health.

[21] Gardiner C.K., Bryan A.D. (2017). Monetary incentive interventions can enhance psychological factors related to fruit and vegetable consumption. Ann. Behav. Med..

[22] Kral T.V.E., Bannon A.L., Moore R.H. (2016). Effects of financial incentives for the purchase of healthy groceries on dietary intake and weight outcomes among older adults: A randomized pilot study. Appetite.

[23] Herman D.R., Harrison G.G., Afifi A.A., Jenks E. (2008). Effect of a targeted subsidy on intake of fruits and vegetables among low-income women in the special supplemental nutrition program for women, infants, and children. Am. J. Public Health.

[24] Anderson J.V., Bybee D.I., Browm R.M., McLean D.F., Garcia E.M., Breer M.L., Schillo B.A. (2001). 5 a day fruit and vegetable intervention improves consumption in a low income population. J. Am. Diet. Assoc..

[25] Perez J., Allen P., Brown M. (2003). Community Supported Agriculture on the Central Coast: The CSA Member Experience.

[26] Wharton C.M., Hughner R.S., MacMillan L., Dumitrescu C. (2015). Community Supported Agriculture programs: A novel venue for theory-based health behavior change interventions. Ecol. Food Nutr..

[27] Northeast Organic Farming Asssociation of Vermont (NOFA-VT) (2016). Fresh & Affordable: Full Report from NOFA-VT’s 2016 Study on Price Competitiveness of Products Purchased Directly from Local Farmers.

[28] Connor D.S. (2003). Community Supported Agriculture Pricing and Promotion Strategies: Lessons from Two Ithaca, NY, Area Farms.

[29] Brehm J.M., Eisenhauer B.W. (2008). Motivations for Participating in Community-Supported Agriculture and their Relationship with Community Attachment and Social Capital. South. Rural Sociol..

[30] McCormack L.A., Laska M.N., Larson N.I., Story M. (2010). Review of the nutritional implications of farmers’ markets and community gardens: A call for evaluation and research efforts. J. Am. Diet. Assoc..

[31] Minaker L.M., Raine K.D., Fisher P., Thompson M.E., Van Loon J., Frank L.D. (2014). Food purchasing from farmers’ markets and Community-Supported Agriculture is associated with reduced weight and better diets in a population-based sample. J. Hunger Environ. Nutr..

[32] O’Kane G. (2012). What is the real cost of our food? Implications for the environment, society and public health nutrition. Public Health Nutr..

[33] Hanson K.L., Garner J., Connor L.M., Jilcott Pitts S.B., McGuirt J., Harris R., Kolodinsky J., Wang W.W., Sitaker M., Ammerman A.S. (2018). Fruit and vegetable preferences and practices may hinder participation in Community-Supported Agriculture among low-income rural families. J. Nutr. Educ. Behav..

[34] Galt R.E., Bradley K., Christensen L., Fake C., Munden-Dixon K., Simpson N., Surls R., Van Soelen Kim J. (2017). What difference does income make for Community Supported Agriculture (CSA) members in California? Comparing lower-income and higher-income households. Agric. Hum. Values.

[35] Niebylski M.L., Redburn K.A., Duhaney T., Campbell N.R. (2015). Healthy food subsidies and unhealthy food taxation: A systematic review of the evidence. Nutrition.

[36] Cotter E.W., Teixeira C., Bontrager A., Horton K., Soriano D. (2017). Low-income adults’ perceptions of farmers’ markets and Community-Supported Agriculture programmes. Public Health Nutr..

[37] Andreatta S., Rhyne M., Dery N. (2008). Lessons learned from advocating CSAs for low-income and food insecure households. South. Rural Sociol..

[38] Hoffman J.A., Agrawal T., Wirth C., Watts C., Adeduntan G., Myles L., Castaneda-Sceppa C. (2012). Farm to family: Increasing access to affordable fruits and vegetables among urban head start families. J. Hunger Environ. Nutr..

[39] Lang K.B. (2010). The changing face of Community-Supported Agriculture. Cult. Agric..

[40] Quandt S.A., Dupuis J., Fish C., D’Agostino R.B. (2013). Feasibility of using a Community-Supported Agriculture program to improve fruit and vegetable inventories and consumption in an underresourced urban community. Prev. Chronic. Dis..

[41] White M.E.A. (2018). The perceived influence of cost-offset Community Supported Agriculture on food access among low-income families. Public Health Nutr..

[42] Hanson K.L., Kolodinsky J., Wang W.W., Morgan E.H., Pitts S.B.J., Ammerman A.S., Sitaker M., Seguin R.A. (2017). Adults and children in low-income households that participate in cost-offset Community Supported Agriculture have high fruit and vegetable consumption. Nutrients.

[43] Condrasky M.D., Williams J.E., Catalano P.M., Griffin S.F. (2011). Development of psychosocial scales for evaluating the impact of a culinary nutrition education program on cooking and healthful eating. J. Nutr. Educ. Behav..

[44] Beydoun M.A., Wang Y.F. (2008). Do nutrition knowledge and beliefs modify the association of socio-economic factors and diet quality among US adults?. Prev. Med..

[45] Robinson-O’Brien R., Neumark-Sztainer D., Hannan P., Burgess-Champoux T., Haines J. (2009). Fruits and vegetables at home: Child and parent perceptions. J. Nutr. Educ..

[46] Thompson F., Subar A., Coulson A., Boushey C., Ferruzzi M. (2013). Dietary Assessment Methodology. Nutrition in the Prevention and Treatment of Disease.

[47] Thompson F.E., Subar A.F., Smith A.F., Midthune D., Radimer K.L., Kahle L.L., Kipnis V. (2002). Fruit and vegetable assessment: Performance of 2 new short instruments and a food frequency questionnaire. J. Am. Diet. Assoc..

[48] Yaroch A.L., Tooze J., Thompson F.E., Blanck H.M., Thompson O.M., Colón-Ramos U., Shaikh A.R., McNutt S., Nebeling L.C. (2012). Evaluation of three short dietary instruments to assess fruit and vegetable intake: The National Cancer Institute’s food attitudes and behaviors survey. J. Acad. Nutr. Diet..

[49] Greene G.W., Resnicow K., Thompson F.E., Peterson K.E., Hurley T.G., Hebert J.R., Toobert D.J., Williams G.C., Elliot D.L., Goldman Sher T. (2008). Correspondence of the NCI Fruit and Vegetable Screener to Repeat 24-H Recalls and Serum Carotenoids in Behavioral Intervention Trials. J. Nutr..

[50] Collins C.E., Watson J., Burrows T. (2010). Measuring dietary intake in children and adolescents in the context of overweight and obesity. Int. J. Obes..

[51] World Health Organization, Food and Agriculture Organization (2004). Fruit and Vegetables for Health. Proceedings of the Report of a Joint FAO/WHO Workshop.

[52] Fred Hutchinson Cancer Research Center Beverage and Snack Questionnaire 2. https://sharedresources.fredhutch.org/documents/beverage-and-snack-questionnaire-2.

[53] Neuhouser M.L., Lilley S., Lund A., Johnson D.B. (2009). Development and validation of a beverage and snack questionnaire for use in evaluation of school nutrition policies. J. Am. Diet. Assoc..

[54] Bickel G., Nord M., Price C., Hamilton W., Cook J. (2000). Guide to Measuring Household Food Security, Revised 2000.

[55] Cohen J.N., Gearhart S., Garland E. (2012). Community Supported Agriculture: A commitment to a healthier diet. J. Hunger Environ. Nutr..

[56] Landis B., Smith T.E., Lairson M., Mckay K., Nelson H., O’Briant J. (2010). Community-Supported Agriculture in the research triangle region of North Carolina: Demographics and effects of membership on household food supply and diet. J. Hunger Environ. Nutr..

[57] Miewald C., Holben D., Hall P. (2012). Role of a food box program in fruit and vegetable consumption and food security. Can. J. Diet. Pract. Res..

[58] Uribe A.L.M., Winham D.M., Wharton C.M. (2012). Community Supported Agriculture membership in Arizona. An exploratory study of food and sustainability behaviours. Appetite.

[59] Vasquez A., Sherwood N.E., Larson N., Story M. (2017). Community-Supported Agriculture as a dietary and health improvement strategy: A narrative review. J. Acad. Nutr. Diet..

[60] Wilkins J.L., Farrell T.J., Rangarajan A. (2015). Linking vegetable preferences, health and local food systems through Community-Supported Agriculture. Public Health Nutr..

[61] Izumi B.T., Higgins C.E., Baron A., Ness S.J., Allan B., Barth E.T., Smith T.M., Pranian K., Frank B. (2018). Feasibility of using a Community-Supported Agriculture program to increase access to and intake of vegetables among federally qualified health center patients. J. Nutr. Educ. Behav..

[62] Houwer J.D., Thomas S., Baeyens F. (2001). Associative learning of likes and dislikes: A review of 25 years of research on human evaluative conditioning. Psychol. Bull..

[63] Cooley J.P., Lass D.A. (1998). Consumer benefits from Community Supported Agriculture membership. Rev. Agric. Econ..

[64] Russell W.S., Zepeda L. (2008). The adaptive consumer: Shifting attitudes, behavior change and CSA membership renewal. Renew. Agric. Food Syst..

[65] Schnell S.M. (2007). Food with a farmer’s face: Community-Supported Agriculture in the United States. Geogr. Rev..

[66] Barbosa L.B., Vasconcelos S.M.L., Correia L.O.D., Ferreira R.C. (2016). Nutrition knowledge assessment studies in adults: A systematic review. Cien. Saude Colet..

[67] Wang D.D., Leung C.W., Li Y.P., Ding E.L., Chiuve S.E., Hu F.B., Willett W.C. (2014). Trends in dietary quality among adults in the United States, 1999 through 2010. JAMA Intern. Med..

